# A descriptive study of fatal drownings among children and adolescents in the United States, with a focus on retention pond deaths, 2004–2020

**DOI:** 10.1371/journal.pgph.0004106

**Published:** 2025-01-15

**Authors:** Saroj Bista, Nichole L. Michaels

**Affiliations:** 1 Center for Injury Research and Policy, The Abigail Wexner Research Institute at Nationwide Children’s Hospital, Columbus, Ohio, United States of America; 2 Department of Pediatrics, College of Medicine, The Ohio State University, Columbus, Ohio, United States of America; Yale University School of Medicine, UNITED STATES OF AMERICA

## Abstract

The objective of this study was to characterize fatal drownings among children and adolescents, with a focus on retention pond drownings, and identify risk factors for these fatalities using child death review data. We acquired 2004–2020 National Fatality Review-Case Reporting System data for drowning deaths among youth 0–19 years. Retention pond drownings were identified through case narratives. We analyzed demographics, primary caregiver, supervisor, death investigation, and drowning-related variables across age groups (<1, 1–4, 5–9, 10–14, and 15–19 years) using either the Chi-square test or Fisher’s Exact test, as appropriate, with a p-value of <0.05 indicating statistical significance. Of 7,539 drowning deaths reported during the study duration, 265 deaths occurred in retention ponds. Children 1–4 years comprised a greater proportion of retention pond drowning deaths (59.3%) compared to overall drowning deaths (50.9%) in this age group. In 51.3% of retention pond drowning deaths among children <5 years, supervision was needed but not provided. Most (80.4%) retention pond-related deaths among children <5 years and 50.7% of deaths among children 5–19 years were attributed to child neglect, poor or absent supervision, or exposure to hazards. Among all decedents who drowned in retention ponds, 19.1% were found to have a disability or chronic illness. Most retention ponds lacked local ordinances regulating water access (83.5%) and did not have barriers or protection (66.1%) or warning signs (82.0%). Younger children with poor or absent supervision who cannot swim are at high risk of drowning. Retention pond drownings are not infrequent, and most locations lack ordinances regulating water access or requiring barriers, such as fences. Greater efforts are needed to address this common hazard and environmental and policy strategies should be implemented to prevent future deaths.

## Introduction

Drowning is a leading cause of death for children in the United States (US) and globally [1,2]. Annually, an estimated 1,056 youth aged 0–19 years die from unintentional drowning in the US, equating to an average of 3 deaths per day [1]. Children 1–4 years old are at particularly high risk of drowning, with an age-adjusted mortality rate of 3.1 per 100,000 US children [3]. Infants younger than 1 year of age and adolescents and young adults aged 15–24 years have the second-highest age-adjusted fatal drowning rate of 1.1 per 100,000 US population [3]. Among children aged 5–14 years, drowning is the second leading cause of unintentional injury death, after motor vehicle crashes [4]. For two decades, drowning fatalities among children and adolescents in the US declined steadily, with a 38% decrease in the drowning death rate among children 0–17 years from 1999 to 2019 (crude rates: 1.6 and 1.0 deaths per 100,000 population in 1999 and 2019, respectively) [5]. However, between 2019 and 2022, unintentional drowning rates in the US increased significantly [3]. This increase was particularly pronounced among children 1–4 years, with drowning fatality rates increasing by 28%, from 2.4 deaths per 100,000 population in 2019 to 3.1 deaths per 100,000 population in 2022 [3].

Not all youth are at equal risk of drowning. Research identifies significant racial/ethnic disparities in drowning rates, with certain groups, such as American Indian or Alaska Native and Black or African-American individuals, being at higher risk [6–8]. These racial disparities are multifaceted and have roots in systemic racism, such as fewer opportunities for swimming lessons and a lack of access to safe swimming facilities [9,10]. There are also age-specific environmental factors that increase drowning risk. Among young children, one of the greatest risk factors is the lack of barriers to prevent unanticipated, unsupervised access to water. Bathtubs and buckets are common drowning locations for infants, while the majority of children 1–4 years who drown do so in swimming pools, and children aged 5 to 19 years most frequently drown in natural freshwater such as rivers, ponds, or lakes [8]. Among older children and adolescents, overestimation of skills, poor water competency, underestimation of dangerous situations, high-risk and impulsive behaviors, and substance use can increase drowning risk [11–14]. Children who have disabilities may also be at greater risk for drowning, particularly children with autism spectrum disorder. There may be additional risk factors for drowning that have not yet been identified [15,16].

**Fig 1:**
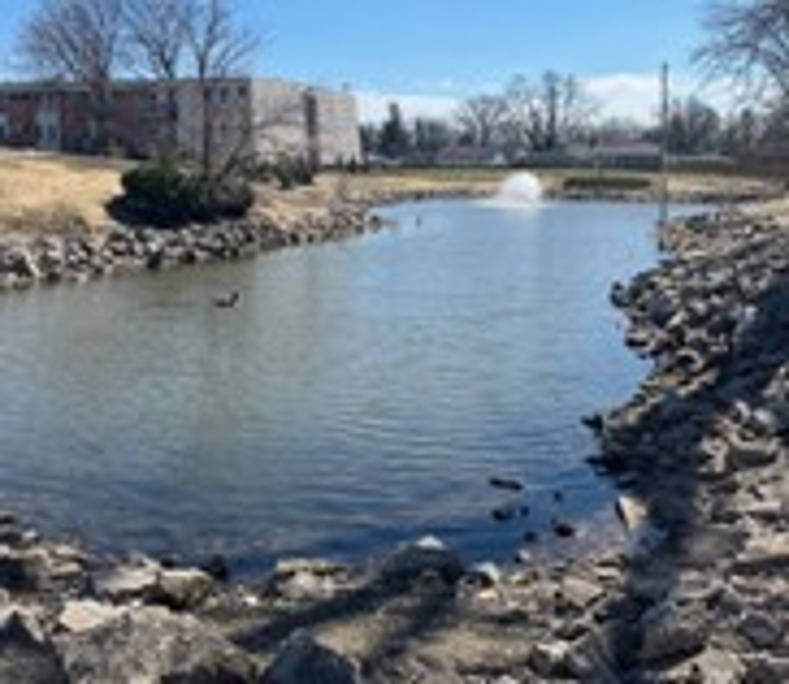
Retention pond, Franklin County, Ohio. Photo by author Michaels, NL.

While many environments are well-recognized for their drowning risk, some may be less obvious. Retention ponds, also called retention basins, are man-made stormwater management ponds designed primarily to attenuate surface runoff during rainfall (Fig 1). Retention ponds are prevalent throughout the US. In suburban areas they are often found in housing subdivisions or near businesses and in rural areas water captured in retention ponds may be used for irrigation. Retention ponds may have fountains or other aesthetic features and are not typically surrounded by fencing or other barriers. While the authors were not able to identify any prior peer-reviewed research studies describing retention pond drownings, news reports indicate that numerous communities have identified these environments as hazardous, particularly to young children [17–23]. One urban fatality review board found that 70% of children in immigrant families who died from drowning did so in open water locations, including retention ponds [21].

Existing research has quantified some aspects of pediatric drownings, such as demographics and location data; however, there is little recently published data describing the circumstances of these deaths in detail [1,6,24–31]. Databases commonly used for drowning research include: the Centers for Disease Control and Prevention’s Wide-ranging ONline Data for Epidemiologic Research (WONDER) [1,24]; inpatient/hospitalization databases [29,31]; and U.S. Consumer Product Safety Commission data [26,29]. Although these databases provide important insights into pediatric drowning, they are not optimized to capture comprehensive data surrounding the circumstances of the deaths. The National Fatality Review-Case Reporting System (NFR-CRS) provides a unique opportunity to comprehensively assess fatal drownings among children on a national level, allowing us to develop a more nuanced understanding of the circumstances surrounding these deaths. It also allows us to investigate pediatric drowning deaths that occur in retention ponds/basins. Identifying risk factors for these fatalities can inform policy and prevention strategies to prevent future injuries and deaths.

Our study objectives were to:

1. Describe the circumstances of all drowning deaths among children and adolescents ages 0–19 years that were reported to the NFR-CRS.
2. Identify and characterize retention pond/basin drowning deaths among this population.

## Methods

### Data source and study population

This study utilized data from the US National Center for Fatality Review and Prevention (National Center). The National Center developed NFR-CRS, a web-based case reporting tool with a standard format for collecting, summarizing, and reporting information shared during the child death review (CDR) process. In 2020, the NFR-CRS received data from 46 states [32]. The NFR-CRS currently collects data on more than 2,600 variables related to demographics, circumstances of death, and death investigation outcomes and actions [33–35]. Local protocols and resources determine voluntary CDR reporting to the NFR-CRS. Further details on the technical aspects of the database, its strengths, limitations, and recent data quality efforts can be found elsewhere [33,36]. Our study utilized all available data, focusing on fatalities among children and adolescents aged 0 to 19 years that occurred between 2004 and 2020, for which the primary cause of death was identified as “from an external cause” and the external cause of injury was listed as “drowning” in the NFR-CRS. To identify and characterize retention pond drowning deaths, we utilized multiple variables, as the NFR-CRS does not contain a specific retention pond drowning variable. The National Center team reviewed narratives of the deaths in which the drowning location was listed as “other, specify” (Question H3b) or “open water/pond” (Question H3b), and the open water location was identified as “pond,” “quarry or gravel pit,” “canal/drainage ditch,” or “unknown” (Question H3c). Narratives were not reviewed for drownings in locations categorized as “open water/pond” (Question H3b) if the open water location was further identified as “lake,” “river,” “creek,” or “ocean” (Question H3c). Due to restrictions on sharing narrative data, the National Center team conducted the case narrative review. Search terms and phrases used included: “man made pond,” “manmade pond,” “man-made pond,” “man-made lagoon,” “retention pond,” “farm pond,” “koi pond,” “decorative pond,” “decorative fountain,” “yard pond,” “landscape pond,” “drainage canal,” “drainage tube,” “drainage pond.” Of the 266 children identified by the National Center team, one child was removed by the study team due to reporting inconsistencies.

### Study variables

NFR-CRS variables requested for this study include the following categories: child, primary caregiver, and supervisor demographics, incident and death investigation information, and drowning-related variables. Child supervision at the time of the incident was determined by CDR teams based on NFR-CRS criteria that take into consideration the supervisor’s physical proximity to the child, whether the supervisor could see or hear the child, and the child’s need for supervision based on their developmental age and other circumstances. For example, CDR coding guidelines indicate that if the child is <6 years, the supervisor should be able to see or hear the child, while older children may need less close supervision, depending on circumstances. The NFR-CRS includes two separate variables for race and ethnicity. These variables are important to the study because they relate to past health inequalities that have affected child mortality [37].

### Statistical analysis

Because risk factors for drowning vary by age, characteristics of the decedents and circumstances of their deaths were analyzed using five age categories: <1, 1–4, 5–9, 10–14, and 15–19 years. However, due to the small sample size in the retention pond deaths, only two age groups, <5 and 5–19 years, were used. Basic descriptive statistics are reported and age groups were compared using either the Chi-square test or Fisher’s Exact test, as appropriate, with a p-value of <0.05 indicating statistical significance. Values that were unknown/missing or not applicable are reported but excluded from the denominator when calculating percentages. Cell counts ≤5 were suppressed per National Center data-use protocols to prevent inadvertent identification of decedents. Variables with ≥50% missing data are not reported. Some NFR-CRS variables, such as primary caregiver’s/supervisor’s relation to child, incident place, etc., were combined for analysis purposes to account for sample size limitations. The authors’ institutional review board exempted this study from review. All analyses were performed using SAS 9.4 (SAS Institute Inc., Cary, NC).

## Results

### Overall decedent characteristics and incident circumstances

There were 7,539 children and adolescents aged 0–19 years with drowning as the primary cause of death reported to the NFR-CRS during 2004–2020 (Table 1). Most decedents were aged 1–4 years (50.9%), male (69.6%), and White (67.8%). Overall, 17.5% of all children and 31.1% of children 5–9 years were identified as having a disability or chronic illness. The proportion of males and children with disability or chronic illness increased with age. The race distribution differed significantly by age (*p-value* <0.001). More than three-fourths (76.3%) of children aged 1–4 years old were White and 16.0% were Black. However, among children 5–9 years and 10–14 years, the percentage of White versus Black decedents was 56.1% vs 35.2% and 54.4% vs 37.8%, respectively.

**Table 1:** Selected demographic and social characteristics of fatal child and adolescent drownings by age, National Fatality Review Case Reporting System, 2004–2020.

Characteristics	Number (Percent)^a^						*p-value*
	Total	Age, Years					
		<1	1–4	5–9	10–14	15–19	
**Child (decedents)**	7539 (100.0)	528 (7.0)	3836 (50.9)	1141 (15.1)	867 (11.5)	1167 (15.5)	
Sex							**<0.001**
Male	5220 (69.6)	293 (56.0)	2497 (65.4)	813 (71.4)	621 (72.5)	996 (85.6)	
Female	2278 (30.4)	230 (44.0)	1319 (34.6)	326 (28.6)	236 (27.5)	167 (14.4)	
Missing/Unknown (*n* = 41)^b^							
Race							**<0.001**
White	4812 (67.8)	305 (62.4)	2745 (76.3)	608 (56.1)	443 (54.4)	711 (63.9)	
Black	1701 (24.0)	128 (26.2)	574 (16.0)	382 (35.2)	308 (37.8)	309 (27.8)	
American Indian and Alaska Native	170 (2.4)	24 (4.9)	87 (2.4)	18 (1.7)	20 (2.5)	21 (1.9)	
Asian	226 (3.2)	—	97 (2.7)	—	22 (2.7)	50 (4.5)	
Native Hawaiian and Pacific Islander	41 (0.6)	—	14 (0.4)	—	8 (1.0)	9 (0.8)	
Two or more races	146 (2.1)	17 (3.5)	79 (2.2)	24 (2.2)	14 (1.7)	12 (1.1)	
Missing/Unknown (*n* = 443)^b^							
Ethnicity							**<0.001**
Hispanic	1396 (20.3)	136 (28.5)	740 (21.1)	169 (16.1)	138 (17.7)	213 (19.9)	
Not Hispanic	5492 (79.7)	342 (71.6)	2763 (78.9)	884 (84.0)	643 (82.3)	860 (80.2)	
Missing/Unknown (*n* = 651)^b^							
Disability or chronic illness							**<0.001**
Yes	936 (17.5)	15 (3.9)	264 (9.4)	257 (31.1)	201 (34.2)	199 (27.7)	
No	4410 (82.5)	373 (96.1)	2561 (90.7)	569 (68.9)	387 (65.8)	520 (72.3)	
Missing/Unknown (*n* = 2193)^b^							
**Primary caregiver**							
Primary caregivers’ relation to child							**<0.001**
Biological parent	6074 (87.7)	484 (95.3)	3270 (90.8)	919 (87.2)	661 (85.6)	740 (74.5)	
Grandparent/Sibling/Other relative	325 (4.7)	10 (2.0)	152 (4.2)	63 (6.0)	42 (5.4)	58 (5.8)	
Other^c^	287 (4.1)	10 (2.0)	133 (3.7)	46 (4.4)	39 (5.1)	59 (5.9)	
Self	142 (2.0)	—	8 (0.2)	—	15 (1.9)	116 (11.7)	
Adoptive parent/Stepparent	101 (1.5)	—	38 (1.1)	—	15 (1.9)	21 (2.1)	
Missing/Unknown (*n* = 610)^b^							
Age, years (mean ± SD) (*n’* = 4455)	32 (9.9)	26.2 (6.6)	30.5 (8.6)	33.8 (8.8)	38.7 (10.2)	36.8 (13.6)	**<0.001**
Sex							**<0.001**
Male	1154 (17.1)	43 (8.6)	492 (14.0)	148 (14.4)	169 (22.6)	302 (31.9)	
Female	5595 (82.9)	459 (91.4)	3033 (86.0)	878 (85.6)	579 (77.4)	646 (68.1)	
Missing/Unknown (*n* = 790)^b^							
Ability to understand English							**0.001**
Yes	5003 (94.7)	415 (97.7)	2711 (95.2)	777 (93.1)	526 (93.4)	574 (93.2)	
No	283 (5.4)	10 (2.4)	136 (4.8)	58 (7.0)	37 (6.6)	42 (6.8)	
Missing/Unknown (*n* = 2253)^b^							
Perpetrator of maltreatment							**<0.001**
Yes	962 (25.2)	101 (32.3)	502 (23.1)	174 (28.6)	106 (29.7)	79 (21.6)	
No	2857 (74.8)	212 (67.7)	1672 (76.9)	435 (71.4)	251 (70.3)	287 (78.4)	
Missing/Unknown (*n* = 3720)^b^							
**Supervisor** ^d^							
Supervisor relationship to child							**<0.001**
Biological parent	3822 (67.6)	414 (85.9)	2394 (70.3)	616 (63.9)	289 (52.9)	109 (42.4)	
Grandparent/Sibling/Other relative	1032 (18.2)	35 (7.3)	656 (19.3)	189 (19.6)	112 (20.5)	40 (15.6)	
Other^e^	644 (11.4)	27 (5.6)	300 (8.8)	124 (12.9)	107 (19.6)	86 (33.5)	
Adoptive parent/Stepparent	92 (1.6)	—	—	29 (3.0)	20 (3.7)	9 (3.5)	
Other^f^	59 (1.0)	—	—	6 (0.6)	18 (3.3)	13 (5.1)	
Self	6 (0.1)	—	—	0 (0.0)	0 (0.0)	0 (0.0)	
Missing/Unknown (*n* = 393)^b^							
Age, years (mean ± SD) (*n’* = 3716)	32.4 (11.6)	26.9 (8.1)	32 (11.6)	33.8 (11.4)	37.4 (11.6)	39.8 (12.7)	**<0.001**
Sex							**<0.001**
Male	1634 (30.2)	92 (19.4)	936 (28.3)	275 (30)	209 (42.2)	122 (58.1)	
Female	3776 (69.8)	383 (80.6)	2376 (71.7)	643 (70)	286 (57.8)	88 (41.9)	
Missing/Unknown (*n* = 638)^b^							
Ability to understand English							0.15
Yes	4312 (95.0)	392 (96.8)	2609 (94.8)	736 (93.8)	394 (96.1)	181 (95.8)	
No	228 (5.0)	13 (3.2)	142 (5.2)	49 (6.2)	16 (3.9)	8 (4.2)	
Missing/Unknown (*n* = 1508)^b^							

—, per-state data-use agreements, counts of n ≤ 5 were suppressed. Bold indicates statistical significance.

^a^Column percentage may not sum to 100.0% because of rounding error.

^b^Unknown and missing values were omitted from the denominator when calculating percentages.

^c^Other included foster parent, parents’ partner, friend, institutional staff, or other.

^d^Supervisor information was collected if a child had supervision at the time of incident leading to death, had no supervision, but needed, or supervision information could not be determined.

^e^Other included foster parent, parents’ partner, friend, acquaintance, babysitter, or other.

^f^Other included hospital, institutional staff, or licensed childcare worker.

*n’*, effective sample size; SD, standard deviation.

Biological parents were identified as primary caregivers for nearly 9 out of 10 children (87.7%). Most primary caregivers were female (82.9%), with a mean age of 32 years (standard deviation (SD): 9.9). The primary caregiver for a one-quarter of children (25.2%) had prior documented history of being the perpetrator of child maltreatment.

Biological parents were identified as a supervisor at the time of the incident for 67.6% of children, followed by relatives including grandparent, sibling, or other relative (18.2%). Females were a supervisor in 69.8% of incidents, and the mean age of supervisors was 32.4 years (SD: 11.6). In 40.1% of deaths, the CDR identified the decedent as needing supervision, but found supervision was not provided (Table 2). More than three-quarters (78.0%) of deaths were attributed to abuse, neglect, poor/absent supervision, or exposure to hazards, and almost one-quarter (23.4%) of adolescents 15–19 years were documented to have used drugs or alcohol at the time of the incident leading to death. Overall, 46.4% of drowning deaths occurred in pools, hot tubs, or spas, while 32.9% occurred in open water, including retention ponds (Table 3).

**Table 2:** Child and adolescent drowning incident and investigative characteristics by age, National Fatality Review Case Reporting System, 2004–2020.

Characteristics	Number (Percent)^a^						*p-value*
	Total	Age, Years					
		<1	1–4	5–9	10–14	15–19	
Total	7539 (100.0)	528 (7.0)	3836 (50.9)	1141 (15.1)	867 (11.5)	1167 (15.5)	
Incident place							**<0.001**
Child’s home	2596 (35.9)	415 (81.2)	1722 (46.9)	229 (20.9)	127 (15.1)	103 (9.2)	
Relative’s home	769 (10.6)	23 (4.5)	640 (17.4)	72 (6.6)	25 (3.0)	9 (0.8)	
Friend’s home	418 (5.8)	—	—	95 (8.7)	32 (3.8)	31 (2.8)	
Other recreation area	1156 (16.0)	—	—	235 (21.4)	269 (32.1)	436 (39.1)	
Other^d^	2297 (31.7)	64 (12.5)	845 (23.0)	466 (42.5)	386 (46.0)	536 (48.1)	
Missing/Unknown (*n* = 303)^b^							
Incident area							**<0.001**
Urban	1788 (28.4)	196 (43.4)	925 (29.3)	287 (29.2)	184 (25.0)	196 (20.2)	
Suburban	2175 (34.6)	161 (35.6)	1174 (37.2)	330 (33.6)	236 (32.0)	274 (28.3)	
Rural	2277 (36.2)	—	1039 (33.0)	—	307 (41.7)	479 (49.4)	
Frontier	55 (0.9)	—	15 (0.5)	—	10 (1.4)	21 (2.2)	
Missing/Unknown (*n* = 1244)^b^							
Supervision at time of incident							**<0.001**
No, not needed	1106 (15.5)	16 (3.1)	82 (2.2)	54 (5.0)	196 (23.8)	758 (69.8)	
No, but needed	2869 (40.1)	270 (52.9)	1868 (51.1)	437 (40.6)	205 (24.9)	89 (8.2)	
Yes	2844 (39.8)	214 (42.0)	1566 (42.8)	524 (48.7)	349 (42.5)	191 (17.6)	
Time before incident supervisor saw child							
Child in sight	805 (42.2)	69 (46.0)	251 (24.4)	206 (58.4)	174 (72.5)	105 (78.4)	
Time in min (mean ± SD) (*n’* = 1103)	24.4 (55.6)	24.2 (49.2)	24.5 (57.6)	24.4 (56.8)	24.2 (41.7)	22.7 (38.9)	0.75
Time in min (median, min-max) (*n’* = 1103)	10 (0–660)	10 (1–360)	10 (0–660)	10 (1–480)	10 (1–240)	10 (2–180)	
Missing/Unknown (*n* = 936)^b^							
Unable to determine	335 (4.7)	10 (2.0)	143 (3.9)	62 (5.8)	72 (8.8)	48 (4.4)	
Missing/Unknown (*n* = 385)^b^							
*(*n’* = 6048, 84.5%)							
Supervisor asleep or impaired^e^							**<0.001**
Yes	2471 (55.3)	265 (66.8)	1627 (58.6)	383 (51.7)	143 (37.5)	53 (31.0)	
No	1997 (44.7)	132 (33.3)	1151 (41.4)	358 (48.3)	238 (62.5)	118 (69)	
Missing/Unknown (*n* = 1580)^b^							
Supervisor impaired^e^							**<0.001**
Yes^f^	2166 (51.7)	228 (60.8)	1395 (53.4)	358 (51.4)	137 (38.8)	48 (30.8)	
Distracted	1395 (64.4)	112 (49.1)	964 (69.1)	240 (67.0)	62 (45.3)	17 (35.4)	**<0.001**
Absent	680 (31.4)	83 (36.4)	396 (28.4)	116 (32.4)	64 (46.7)	21 (43.8)	**<0.001**
Drug impaired	174 (8.0)	40 (17.5)	116 (8.3)	15 (4.2)	—	—	**<0.001**
Alcohol impaired	136 (6.3)	26 (11.4)	76 (5.5)	19 (5.3)	—	—	**0.011**
Impaired by illness/disability	46 (2.1)	14 (6.4)	23 (1.7)	7 (2.0)	—	—	**0.003**
Impaired - other^g^	153 (7.1)	21 (9.2)	87 (6.2)	23 (6.4)	14 (10.2)	8 (16.7)	**0.017**
No	2027 (48.3)	147 (39.2)	1218 (46.6)	338 (48.6)	216 (61.2)	108 (69.2)	
Missing/Unknown (*n* = 1855)^b^							
Incident date same as death date							0.13
Yes	5472 (77.7)	402 (79.8)	2781 (77.0)	831 (78.4)	617 (75.9)	841 (79.9)	
No	1572 (22.3)	102 (20.2)	833 (23.1)	229 (21.6)	196 (24.1)	212 (20.1)	
Missing/Unknown (*n* = 495)^b^							
Incident time of day							**<0.001**
8 AM–11 AM	703 (12.5)	80 (21.1)	431 (14.7)	69 (7.9)	49 (7.9)	74 (9.1)	
12 PM–7 PM	3594 (63.9)	182 (47.9)	1851 (63.0)	608 (70.0)	419 (67.5)	534 (65.4)	
8 PM–11 PM	824 (14.7)	63 (16.6)	425 (14.5)	123 (14.2)	92 (14.8)	121 (14.8)	
12 AM–7 AM	503 (8.9)	55 (14.5)	231 (7.9)	69 (7.9)	61 (9.8)	87 (10.7)	
Missing/Unknown (*n* = 1915)^b^							
Resuscitation attempted							**<0.001**
Yes	5732 (91.9)	439 (97.1)	3205 (97.4)	877 (91.8)	572 (82.9)	639 (75.1)	
No	507 (8.1)	13 (2.9)	86 (2.6)	78 (8.2)	118 (17.1)	212 (24.9)	
N/A (*n* = 47)^c^							
Missing/Unknown (*n* = 1253)^b^							
Child had used drugs or alcohol at time of incident leading to death							**<0.001**
Yes	233 (6.4)	—	6 (0.4)	—	42 (7.6)	178 (23.4)	
No	3438 (93.7)	—	1538 (99.6)	—	509 (92.4)	583 (76.6)	
N/A (*n* = 2549)^c^							
Missing/Unknown (*n* = 1319)^b^							
Total deaths at incident							**<0.001**
1 child	6491 (94.4)	485 (98)	3384 (96.2)	941 (89.7)	727 (91.0)	954 (94.1)	
2 or more children	384 (5.6)	10 (2.0)	134 (3.8)	108 (10.3)	72 (9.0)	60 (5.9)	
Missing/Unknown (*n* = 664)^b^							
Toxicology testing performed							**<0.001**
No	1361 (24.3)	70 (17)	763 (26.7)	245 (29.3)	152 (24.4)	131 (15.1)	
Yes	4236 (75.7)	342 (83)	2095 (73.3)	590 (70.7)	471 (75.6)	738 (84.9)	
Results^f^							
Negative	3447 (81.4)	285 (83.3)	1847 (88.2)	499 (84.6)	350 (74.3)	466 (63.1)	**<0.001**
Alcohol	131 (3.1)	—	6 (0.3)	—	26 (5.5)	97 (13.1)	**<0.001**
Marijuana	125 (3.0)	—	—	—	15 (3.2)	104 (14.1)	**<0.001**
Cocaine/Methamphetamine	29 (0.7)	—	7 (0.3)	—	—	14 (1.9)	**<0.001**
Abnormally high Rx/OTC drug	20 (0.5)	0 (0.0)	—	—	—	11 (1.5)	**0.001**
Opioids	19 (0.5)	—	7 (0.3)	—	—	8 (1.1)	0.08
Other^h^	275 (6.5)	15 (4.4)	93 (4.4)	36 (6.1)	49 (10.4)	82 (11.1)	**<0.001**
Missing/Unknown (*n* = 1942)^b^							
Child abuse/neglect/poor or absent supervision/exposure to hazards contributing to death							**<0.001**
Yes/probably	4305 (71.2)	428 (92.4)	2780 (83.4)	667 (73.9)	299 (48.7)	131 (17.9)	**<0.001**
Child abuse	109 (2.5)	32 (7.5)	56 (2.0)	17 (2.6)	—	—	
Child neglect	617 (14.4)	125 (29.4)	387 (14.0)	69 (10.4)	27 (9.0)	9 (6.9)	
Poor/absent supervision	3343 (78.0)	256 (60.2)	2199 (79.5)	538 (81.2)	248 (82.9)	102 (77.9)	
Exposure to hazards	216 (5.0)	12 (2.8)	125 (4.5)	39 (5.9)	—	—	
Missing/Unknown (*n* = 20)^b^							
No	1740 (28.8)	35 (7.6)	552 (16.6)	236 (26.1)	315 (51.3)	602 (82.1)	
Missing/Unknown (*n* = 1494)^b^							
Child protective services (CPS) action							**<0.001**
Yes	1839 (36.7)	253 (65.9)	1190 (44.1)	275 (35.9)	92 (17.5)	29 (4.6)	
No	3166 (63.3)	131 (34.1)	1509 (55.9)	492 (64.2)	435 (82.5)	599 (95.4)	
N/A (*n* = 584)^c^							
Missing/Unknown (*n* = 1950)^b^							

—, per-state data-use agreements, counts of n ≤ 5 were suppressed. Bold indicates statistical significance.

^a^Column percentage may not sum to 100.0% because of rounding error.

^b^Unknown and missing values were omitted from the denominator when calculating percentages.

^c^Not applicable values were reported but omitted from the denominator when calculating percentages.

^d^Other included foster care home, group home, day care center, day care home, farm, school, Indian reservation, military installation, sidewalk, roadway, driveway, other parking area, hospital, multiple places, or other location such as rental place, Airbnb, apartment complex, river, lake, canal, campground, etc.

^e^Information collected if a child had supervision at the time of incident leading to death, had no supervision, but needed, or supervision information could not be determined.

^f^Not mutually exclusive categories.

^g^Other included in the bathroom, inside home, doing some chores, fishing, etc.

^h^Other included caffeine, benzodiazepine, barbiturates, carbon monoxide, inhalants, nicotine, etc.

*n’*, effective sample size; SD, standard deviation; N/A, not applicable; Rx, prescription; OTC, over the counter.

**Table 3:** Child and adolescent drowning characteristics by age, National Fatality Review Case Reporting System, 2004–2020.

Characteristics	Number (Percent)^a^						*p-value*
	Total	Age, Years					
		<1	1–4	5–9	10–14	15–19	
Total	7539 (100.0)	528 (7.0)	3836 (50.9)	1141 (15.1)	867 (11.5)	1167 (15.5)	
Child last seen							**<0.001**
In water	2099 (31.4)	64 (12.9)	443 (13.2)	433 (42.0)	456 (58.5)	703 (68.6)	
In house	1489 (22.2)	74 (15.0)	1229 (36.5)	121 (11.7)	29 (3.7)	36 (3.5)	
Near water	806 (12.0)	—	444 (13.2)	170 (16.5)	—	92 (9.0)	
In yard	624 (9.3)	—	527 (15.7)	64 (6.2)	—	10 (1.0)	
In bathroom/tub	533 (8.0)	191 (38.6)	204 (6.1)	53 (5.1)	52 (6.7)	33 (3.2)	
Other^d^	1144 (17.1)	153 (30.9)	516 (15.3)	191 (18.5)	133 (17.1)	151 (14.7)	
Missing/Unknown (*n* = 844)^b^							
Drowning location							**<0.001**
Open water/pond	2405 (32.9)	17 (3.3)	648 (17.4)	417 (37.7)	492 (58.4)	831 (74.3)	
Open water place							**<0.001**
Lake	835 (35.1)	—	—	143 (34.5)	167 (34.4)	356 (43.5)	
River	590 (24.8)	—	—	96 (23.2)	160 (33)	234 (28.6)	
Pond	509 (21.4)	6 (35.3)	256 (39.6)	93 (22.5)	66 (13.6)	88 (10.7)	
Creek	197 (8.3)	—	61 (9.4)	—	47 (9.7)	46 (5.6)	
Ocean	122 (5.1)	0 (0.0)	13 (2.0)	26 (6.3)	26 (5.4)	57 (7.0)	
Canal/drainage ditch	104 (4.4)	0 (0.0)	54 (8.4)	14 (3.4)	13 (2.7)	23 (2.8)	
Quarry or gravel pit	24 (1.0)	—	—	—	6 (1.2)	15 (1.8)	
Missing/Unknown (*n* = 24)^b^							
Pool, hot tub, spa	3394 (46.4)	52 (10.1)	2451 (65.7)	517 (46.8)	215 (25.5)	159 (14.2)	
Pool type							**<0.001**
In-ground	2072 (70.2)	35 (68.6)	1356 (63.8)	408 (87.9)	147 (86.5)	126 (90.7)	
Above-ground	789 (26.7)	8 (15.7)	698 (32.8)	51 (11.0)	21 (12.4)	11 (7.9)	
Hot tub, spa	55 (1.9)	0 (0.0)	50 (2.4)	—	—	—	
Wading	35 (1.2)	8 (15.7)	23 (1.1)	—	—	—	
Missing/Unknown (*n* = 443)^b^							
Pool ownership							**<0.001**
Private	2668 (88.1)	—	2069 (94.1)	—	125 (69.4)	83 (61.9)	
Public	362 (12)	—	130 (5.9)	—	55 (30.6)	51 (38.1)	
Missing/Unknown^b^							
Bathtub	973 (13.3)	377 (73.4)	368 (9.9)	95 (8.6)	76 (9.0)	57 (5.1)	
Other^e^	540 (7.4)	68 (13.2)	264 (7.1)	77 (7.0)	59 (7.0)	72 (6.4)	
Missing/Unknown (*n* = 227)^b^							
Floatation device^f^							**0.002**
Yes	192 (4.4)	—	83 (3.6)	45 (6.4)	34 (6.3)	—	
No	4215 (95.6)	—	2246 (96.4)	657 (93.6)	508 (93.7)	—	
N/A (*n* = 505)^c^							
Missing/Unknown (*n* = 1452)^b^							
Child able to swim^f^							**<0.001**
Yes	836 (20.0)	—	—	127 (20.9)	235 (50.4)	432 (66.3)	
No	3345 (80.0)	—	—	481 (79.1)	231 (49.6)	220 (33.7)	
N/A (*n* = 141)^c^							
Missing/Unknown (*n* = 2042)^b^							
Barriers or protection^f^							**<0.001**
Yes	2137 (44.8)	42 (53.9)	1613 (60.8)	270 (35.7)	117 (21.8)	95 (12.8)	
No	2629 (55.2)	36 (46.2)	1040 (39.2)	486 (64.3)	419 (78.2)	648 (87.2)	
Missing/Unknown (*n* = 1598)^b^							
Warning sign posted^f^							**<0.001**
Yes	623 (29.7)	9 (27.3)	142 (13.2)	163 (41.8)	134 (52.1)	175 (51.0)	
No	1475 (70.3)	24 (72.7)	933 (86.8)	227 (58.2)	123 (47.9)	168 (49.0)	
N/A (*n* = 1256)^c^							
Missing/Unknown (*n* = 3001)^b^							
Lifeguard present^f^							**<0.001**
Yes	159 (4.7)	0 (0.0)	29 (1.8)	46 (7.5)	44 (9.3)	40 (6.1)	
No	3252 (95.3)	24 (100)	1615 (98.2)	565 (92.5)	430 (90.7)	618 (93.9)	
N/A (*n* = 1566)^c^							
Missing/Unknown (*n* = 1387)^b^							
Rescue attempt made^f^							**0.012**
Yes	4058 (83.4)	70 (76.1)	2154 (84.6)	690 (84.6)	505 (81.5)	639 (80.7)	
No	809 (16.6)	22 (23.9)	393 (15.4)	126 (15.4)	115 (18.6)	153 (19.3)	
N/A (*n* = 471)^c^							
Missing/Unknown (*n* = 1026)^b^							

—, per-state data-use agreements, counts of n ≤ 5 were suppressed. Bold indicates statistical significance.

^a^Column percentage may not sum to 100.0% because of rounding error.

^b^Unknown and missing values were omitted from the denominator when calculating percentages.

^c^Not applicable values were reported but omitted from the denominator when calculating percentages.

^d^Other included in car, apartment complex ground, at a wedding, campsite, classroom, church, community center, hotel, etc.

^e^Other included bucket, well/cistern/septic, toilet, canal, ditch, retention pond, kai pond, decorative pond, puddle, rain tub, etc.

^f^Information collected if drowning location was open water/pond, pool, hot tub, spa, other, or unknown; not collected if drowning occurred in bathroom/tub.

N/A, not applicable.

### Retention pond drowning decedent characteristics and incident circumstances

Of the 265 retention pond drowning deaths identified in the dataset, 60.4% were children 1–4 years (Table 4). Among children who drown in retention ponds, 19.1% of all children, nearly one-half (45.2%) of children 5–9 years, and a substantial proportion of older children had a disability or chronic illness (not shown in the table). Supervision was documented as present at the time of the incident for 30.5% of deaths, while in 44.5% of deaths supervision was needed but not provided. Among deaths for which the time since the child was seen by a supervisor was documented, 29.1% of deaths in retention ponds occurred with the child in sight. In 50.3% of retention pond drowning deaths, supervisors were reported to be either asleep or impaired at the time of the incident.

**Table 4:** Child and adolescent retention ponds drowning incident and investigative characteristics by age, National Fatality Review Case Reporting System, 2004–2020.

Characteristics	Number (Percent)^a^			*p-value*
	Total	Age, Years		
		<5	5–19	
Total	265 (100.0)	160 (60.4)	105 (39.6)	
Incident place				**<0.001**
Child’s home	75 (28.6)	64 (40.5)	11 (10.6)	
Relative’s/Friend’s home	41 (15.7)	—	—	
Other^d^	146 (55.7)	—	—	
Missing/Unknown (*n* = —)^b^				
Incident area				0.16
Urban	56 (23.7)	32 (22.2)	24 (26.1)	
Suburban	79 (33.5)	55 (38.2)	24 (26.1)	
Rural/Frontier	101 (42.8)	57 (39.6)	44 (47.8)	
Missing/Unknown (*n* = 29)^b^				
Supervision at time of incident				**<0.001**
No, not needed	52 (20.3)	6 (3.9)	46 (46.0)	
No, needed	114 (44.5)	80 (51.3)	34 (34.0)	
Yes	78 (30.5)	63 (40.4)	15 (15.0)	
Time before incident supervisor saw child				
Child in sight	16 (29.1)	—	—	
Time in min (mean ± SD) (*n’* = 39)	20.5 (27.1)	18.7 (23.3)	30.5 (44.5)	0.53
Time in min (median, min-max) (*n’* = 39)	10 (2–120)	10 (4–120)	17.5 (2–120)	0.35
Missing/Unknown (*n* = 23)^b^				
Unable to determine	12 (4.7)	—	—	
Missing/Unknown (*n* = 9)^b^				
Supervisor asleep or impaired (*n* = 204)^e^				0.27
Yes	76 (50.3)	55 (47.8)	21 (58.3)	
No	75 (49.7)	60 (52.2)	15 (41.7)	
Missing/Unknown (*n* = 53)^b^				
Incident date same as death date				0.86
Yes	207 (80.5)	127 (80.9)	80 (80.0)	
No	50 (19.5)	30 (19.1)	20 (20.0)	
Missing/Unknown (*n* = 8)^b^				
Resuscitation attempted				**<0.001**
Yes	195 (86.7)	136 (95.8)	59 (71.1)	
No	30 (13.3)	6 (4.2)	24 (28.9)	
N/A (*n* = 6)^c^				
Missing/Unknown (*n* = 34)^b^				
Child had used drugs or alcohol at time of incident leading to death				**0.003**
Yes	9 (7.1)	0 (0.0)	9 (13.6)	
No	118 (92.9)	61 (100.0)	57 (86.4)	
N/A (*n* = 108)^c^				
Missing/Unknown (*n* = 30)^b^				
Total deaths at incident				**0.003**
1, child	233 (92.8)	—	—	
2 or more children	18 (7.2)	—	—	
Missing/Unknown (*n* = 14)^b^				
Toxicology testing performed				0.5
No	54 (25.1)	33 (26.8)	21 (22.8)	
Yes	161 (74.9)	90 (73.2)	71 (77.2)	
Results^f^				**0.03**
Negative	135 (86.5)	79 (91.9)	56 (80.0)	
Positive^g^	21 (13.5)	7 (8.1)	14 (20.0)	
Missing/Unknown (*n* = —)^b^				
Missing/Unknown (*n* = 50)^b^				
Child abuse/neglect/poor or absent supervision/exposure to hazards contributing to death				**<0.001**
Yes/probably	149 (70.0)	111 (80.4)	38 (50.7)	0.19
Child neglect	23 (15.4)	—	—	
Poor/absent supervision	118 (79.2)	88 (79.3)	30 (79.0)	
Exposure to hazards	8 (5.4)	—	—	
No	64 (30.1)	27 (19.6)	37 (49.3)	
Missing/Unknown (*n* = 52)^b^				

—, per-state data-use agreements, counts of n ≤ 5 were suppressed. Bold indicates statistical significance.

^a^Column percentage may not sum to 100.0% because of rounding error.

^b^Unknown and missing values were omitted from the denominator when calculating percentages.

^c^Not applicable values were reported but omitted from the denominator when calculating percentages.

^d^Other included foster care home, day care home, farm, school, Indian reservation, sidewalk, roadway, other recreation area, multiple places, or other location such as rental place, Airbnb, apartment complex, river, lake, canal, campground, etc.

^e^Information collected if a child had supervision at the time of incident leading to death, had no supervision, but needed, or supervision information could not be determined.

^f^Mutually exclusive categories.

^g^Positive results for alcohol, marijuana, opioid, or other drug including caffeine, nicotine, etc.

*n’*, effective sample size; SD, standard deviation; N/A, not applicable.

Thirteen percent (13.3%) of children aged 5–19 years who drown in retention ponds had used drugs or alcohol at the time of the incident leading to the death. Of children 5–19 years old with toxicology testing performed, 20% tested positive for any substance (including alcohol, marijuana, opioids, or other drugs such as caffeine or nicotine). This subgroup had a median age of 15.5 years (range: 11–17 years). Child neglect was a contributing or probable cause for 15.4% of retention pond drowning deaths and poor/absent supervision was a contributing or probable cause for 79.3% of deaths among children <5 years and 79.0% of deaths among children 5–19 years.

Among children 5–19 years who drown in retention ponds, most (70.3%) were last seen in or near water before the drowning incident (Table 5). Overall, most children were not able to swim (85.3%). Two-thirds (66.1%) of deaths occurred at retention ponds without barriers or protection and for 83.5% of the deaths, there were no local ordinances regulating access to the water.

**Table 5:** Child and adolescent retention ponds drowning characteristics by age, National Fatality Review Case Reporting System, 2004–2020.

Characteristics	Number (Percent)^a^			*p-value*
	Total	Age, Years		
		<5	5–19	
Total	265 (100.0)	160 (60.4)	105 (39.6)	
Child last seen				**<0.001**
In/Near water	109 (43.1)	38 (25.0)	71 (70.3)	
In yard	62 (24.5)	54 (35.5)	8 (7.9)	
In house	61 (24.1)	49 (32.2)	12 (11.9)	
Other^d^	21 (8.3)	11 (7.2)	10 (9.9)	
Missing/Unknown (*n* = 12)^b^				
Barriers or protection				**0.002**
Yes	79 (33.9)	59 (41.6)	20 (22.0)	
No	154 (66.1)	83 (58.5)	71 (78.0)	
Missing/Unknown (*n* = 32)^b^				
Local ordinance regulating access to water				0.38
Yes	23 (16.6)	12 (14.3)	11 (20.0)	
No	116 (83.5)	72 (85.7)	44 (80.0)	
Missing/Unknown (*n* = 126)^b^				
Child able to swim				**<0.001**
Yes	27 (14.8)	—	—	
No	156 (85.3)	—	—	
N/A (*n* = 10)^c^				
Missing/Unknown (*n* = 72)^b^				
Warning sign posted				**0.006**
Yes	22 (18.0)	8 (10.5)	14 (30.4)	
No	100 (82.0)	68 (89.5)	32 (69.6)	
N/A (*n* = 36)^c^				
Missing/Unknown (*n* = 107)^b^				
Rescue attempt made				0.40
Yes	146 (71.2)	91 (73.4)	55 (67.9)	
No	59 (28.8)	33 (26.6)	26 (32.10)	
N/A (*n* = 29)^c^				
Missing/Unknown (*n* = 31)^b^				

—, per-state data-use agreements, counts of n ≤ 5 were suppressed. Bold indicates statistical significance.

^a^Column percentage may not sum to 100.0% because of rounding error.

^b^Unknown and missing values were omitted from the denominator when calculating percentages.

^c^Not applicable values were reported but omitted from the denominator when calculating percentages.

^d^Other includes deaths where multiple locations were listed.

N/A, not applicable.

## Discussion

This study characterizes fatal drowning, with an emphasis on drowning in retention ponds, among children and adolescents 0–19 years using comprehensive, multistate data from the NFR-CRS. Over the 17-year study period, an average of 443 drowning deaths were reported to the NFR-CRS each year, and of these, approximately 16 occurred in retention ponds. Overall, 17.5% of all drowning deaths and 19.1% of retention pond drowning deaths involved children with a disability or chronic illness. Although the nature of these conditions cannot be determined from the available data, this finding is consistent with prior research demonstrating increased risk of drowning death among children with conditions such as autism or epilepsy [15,16,38]. Effective supervision is crucial for ensuring the safety of children, particularly those with disabilities or chronic illnesses [38,39]. However, it is concerning that a significant proportion of these children had no supervision or supervision information could not be determined.

Approximately one-half of all drowning deaths and 59.3% of retention pond drowning deaths in this study occurred among children 1–4 years. This is consistent with prior research identifying this as a population at high-risk of drowning [1,24]. Factors that increase risk of drowning among children <5 years include their inquisitive nature and constant movement, and the lack of environmental barriers to prevent unanticipated, unsupervised access to water [11]. Although some drownings happen when parents are present, many occur when a child wanders away from a supervising adult and accesses a backyard pool or other body of water [12].

In the current study, nearly one-half of all drowning deaths (44.0%) and retention pond drowning deaths (47.8%) among children 5–19 occurred in rural regions. These findings highlight differences in drowning fatalities among children in urban and rural areas, which is consistent with prior research and may be attributable to the abundance of natural waterways, distance from essential services, and longer response times in these areas [5,40]. Moreover, it has been observed that people from ethnic-minority backgrounds living in rural areas and new migrant and refugee communities settled in regional and rural locations of high-income countries are at greater risk of drowning [40–42]. Furthermore, the vulnerability of children in immigrant families to fatal drowning, particularly in retention ponds was recently highlighted by one urban fatality review board [21]. The US has a diverse population, with nearly 45.3 million refugees, immigrants, and migrants (RIM) and more than one-quarter (26%) of the 69.7 million children younger than 18 years living with at least one RIM parent [43], making it imperative to assess the risk of drowning in these populations. Although some information on immigrant and minority status is collected by NFR-CRS, it is often missing, making it difficult to assess the risk of drowning in these populations. Future research should focus on studying the drowning risk among RIM populations and identifying linguistically and culturally appropriate prevention strategies.

Overall, White children accounted for greater numbers of drowning deaths compared to Black children in this study. However, 24% of all drowning deaths and more than one-third of drowning deaths among children 5–9 years and 10–14 years occurred among Black children. While NFR-CRS data is not population level and therefore cannot be used to calculate fatality rates, US Census Bureau data estimate that 13.6% of the US population is Black [44], indicating that Black children are likely substantially overrepresented in these drowning deaths. This is consistent with prior research [1,6,24]. These racial disparities are rooted in systemic racism, and may be reflected in differences in swimming ability, access to swim lessons and swimming facilities, reluctance toward swimming, and cultural beliefs [9,10,45–52]. Additional research is needed to fully understand how personal, social, and environmental factors contribute to this racial disparity and how these issues can be addressed to prevent future deaths [6]. In the meantime, barriers to swimming and water competency should be addressed through increased access to convenient no- or low-cost swim lessons that take into consideration the cultural barriers to swimming that exist in some communities [53,54].

Several factors contributed to fatal drowning among children, including lack of supervision, inadequate swimming skills, swimming in places without lifeguards, not using floatation devices, absence of warning signs and barriers (such as fencing), and lack of local ordinances regulating water access at drowning sites. Most states and local municipalities have swimming pool signage and fencing standards. However, few jurisdictions have introduced legislation or implemented regulations requiring fencing and/or signage around retention ponds [55,56]. This difference in regulatory measures highlights the need to implement a more comprehensive and standardized approach to water safety. Alternatives to retention ponds, such as underground stormwater management systems, should also be considered, particularly in residential areas.

Many drownings occurred while the child was unsupervised, however, in some cases a supervisor was present but asleep or impaired. Despite many adolescents 15–19 years reportedly being able to swim, they had the second highest proportion of fatal drowning. One-quarter of teens in this age group also had used alcohol or drugs at the time of the incident leading to death. Although drowning is one of the leading causes of injuries among adolescents, there is a lack of interventions specifically targeted towards them [57]. Prevention efforts could be aided by identifying and addressing substance use among this age group.

Overall, these findings reinforce the recommendations of the American Academy of Pediatrics, the National Drowning Prevention Alliance, and the US National Water Safety Action Plan 2023–2032 that multiple strategies and devices should be used constantly and simultaneously to create several layers of protection against child drowning. Active adult supervision and physical barriers are two critical layers of protection, while swim lessons, learning CPR and rescue techniques, having an emergency action plan, community education, legislation, local enforcement, and public awareness are also important [11,58,59]. Physicians, often seen as trusted and reliable sources of information for parents and caregivers, can play an instrumental role in preventing child drowning and bring attention to the dangers posed by overlooked water hazards, such as retention ponds [39]. During routine check-ups, physicians can assess individual risk factors for drowning, such as developmental stages and delays, parent-reported information regarding the child’s swimming ability, and the presence of conditions like epilepsy or autism, and tailor advice to families based on these risks. Additionally, physicians can encourage families to enroll children in swimming lessons/water safety courses and may serve as advocates for legislative measures to regulate access to these bodies of water [11].

There are limitations to the current study. First, variations in CDR processes and potential subjectivity in data interpretation, reporting, and quality assurance procedures between both reporters and states may contribute to bias [36,60]. Second, because NFR-CRS participation is voluntary, not all states participate and not all participating states review all child deaths. Several factors influence case selection, including statutory requirements, lead agency priorities, program capacity, decedent’s residency, cause/manner of death, and medical examiner jurisdiction [32]. Therefore, the data collected is not representative of the population of participating states or the US. However, participation in the NFR-CRS has increased over time [36,60,61] Third, some NFR-CRS variables may have a high frequency of missing or unknown data, which limits analysis and interpretation. For the same reason, we were unable to report several variables of interest, such as whether the parent was a first-generation immigrant. The National Center’s ongoing initiatives to improve data quality are enhancing the database’s ability to capture the circumstances surrounding pediatric fatalities more accurately [62].

Despite these limitations, the NFR-CRS provides comprehensive data on important factors that are not available in other databases [1,24,26,27,29,31]. Using these data, we were able to characterize fatal drownings in retention ponds, an understudied area of research. These detailed data provide an enriched understanding of drowning incidents, and analyses of various risk factors (e.g., age, disability, supervision, environmental factors) highlight key intervention opportunities. This study reveals critical gaps in local ordinances, as evidenced by the lack of barriers and warning signs at most retention ponds where the fatal drownings occurred, and support the need for additional public health interventions. However, the absence of a specific retention pond drowning location variable and reliance on narrative data was a potential limitation, as it is subject to the availability of information, coders’ interpretation, and human error.

## Conclusion

Children aged 1–4 years and males had the highest proportion of fatal drowning. Younger children comprised a greater proportion of retention pond drowning deaths compared to overall drowning deaths. Common risk factors for drowning included: poor or absent supervision; lack of barriers to access pools, retention ponds, and other water sources; children’s disability or chronic illness; and lack of swimming ability. Alcohol or drug use among supervisors and adolescents was a contributing factor for fatal drowning. Most communities lack regulations requiring fencing or other barriers to prevent children from accessing retention ponds, highlighting the need for a more comprehensive and standardized approach to water safety that addresses these frequently overlooked hazards.
